# The signaling function of CHI3L1/YKL‐40 in neuroinflammation and Alzheimer’s disease

**DOI:** 10.1002/alz.085972

**Published:** 2025-01-03

**Authors:** Yu‐Wen Alvin Huang

**Affiliations:** ^1^ Brown University, Providence, RI USA

## Abstract

**Background:**

Chitinase‐3‐like protein 1 (CHI3L1, or YKL‐40) is an important regulator of immunity and, in the brain, is primarily secreted by activated astrocytes and heralds a neurotoxic inflammatory state. While it has been well known as a high‐profile biomarker for Alzheimer’s disease (AD) and inflammatory brain conditions (e.g., aging, trauma and autoimmune disorders), the biological role of CHI3L1 in neuroinflammation and AD pathogenesis remains to be elucidated.

**Method:**

We used a combination of in‐vitro and in‐vivo tools to recapitulate and manipulate CHI3L1 secretion in neuroinflammation, including i) cultures of primary mouse astrocytes and iPSC‐derived human neurons and neural stem cells (NSCs), and ii) established mouse models of astrocyte‐specific *Chi3l1/Chil1* conditional knockout, Alzheimer’s disease (5xFAD) and the autoimmune‐mediated, primary astrocytopathy (neuromyelitis optica, NMO) hallmarked by CHI3L1 induction.

**Result:**

We first confirmed the induction of CHI3L1 and neuroinflammatory features in cultured astrocytes activated by well‐characterized pro‐inflammatory stimuli, including the cytokine IL‐1b, amyloid‐b peptides (Ab42) and the autoantibody targeting astroglial protein aquaporin 4 (AQP4 IgG, as in NMO). Strikingly, astrocyte‐secreted CHI3L1 inhibited proliferation and neuronal differentiation of NSCs, and enhanced Ab production, tau phosphorylation and apoptosis in differentiated neurons *in vitro*. *In vivo*, we analyzed brain tissues from mice overexpressing CHI3L1 or receiving stereotaxic transfer of AQP4 IgG, and observed a significant reduction in adult hippocampal neurogenesis and defective learning behaviors as in 5XFAD mice; such in‐vivo defects, of note, can be rescued by CHI3L1 depletion in astrocytes, as demonstrated in our compound AD transgenic mice of astrocyte‐specific CHI3L1 knockout. We further characterized the molecular mechanism of CHI3L1 by identifying the receptor and downstream pathway in NSCs. Mechanistically, CHI3L1 engages the CRTH2 receptor and dampens β‐catenin signaling, which is critical for neurogenesis. Finally, we showed that this CHI3L1/CRTH2/β‐catenin cascade can be targeted to restore neurogenesis and ameliorate cognitive deficits resulting from neuroinflammation.

**Conclusion:**

Our findings provide the pioneering evidence for the biological role of CHI3L1 in the brain, departing from its long‐regarded role as merely a biomarker. The discoveries we made here would be highly advantageous for the development of much‐needed therapeutics to prevent neuroinflammatory toxicity in AD and relevant brain disorders.

